# Molecular phylogeny and morphology reveal two new species of *Pleurocordyceps luopingensis* sp. nov. (Polycephalomycetaceae) and *Pseudometarhizium cangyuanense* sp. nov. (Clavicipitaceae) from Yunnan, southwestern China

**DOI:** 10.3897/mycokeys.132.185759

**Published:** 2026-05-14

**Authors:** Zuoheng Liu, Hao Wang, Xiaoyan Liu, Pengbin Han, Wenlin Pan, Hong Yu

**Affiliations:** 1 Yunnan Herbal Laboratory, School of Chemistry and Environment, Yunnan Minzu University, Kunming, 650504, Yunnan, China Yunnan Herbal Laboratory, School of Chemistry and Environment, Yunnan Minzu University Kunming China; 2 Yunnan Key Laboratory of Chiral Functional Substance Research and Application, Yunnan Minzu University, Yuehua Street, Kunming 650504, China Yunnan Key Laboratory of Chiral Functional Substance Research and Application, Yunnan Minzu University Kunming China; 3 School of Mathematics and Computer Science, Yunnan Minzu University, Kunming, 650504, Yunnan, China School of Mathematics and Computer Science, Yunnan Minzu University Kunming China; 4 The International Joint Research Center for Sustainable Utilization of Cordyceps Bioresources in China and Southeast Asia, Yunnan University, Kunming 650504, Yunnan China The International Joint Research Center for Sustainable Utilization of Cordyceps Bioresources in China and Southeast Asia, Yunnan University Kunming China

**Keywords:** Morphology, new taxa, phylogenetic analysis, *
Pleurocordyceps
*, *
Pseudometarhizium
*, taxonomy

## Abstract

Entomopathogenic fungi are species-rich, highly diverse, and widely distributed worldwide. The majority of entomopathogenic fungi belong to the families Clavicipitaceae, Cordycipitaceae, Ophiocordycipitaceae, and Polycephalomycetaceae. However, species of Polycephalomycetaceae and Clavicipitaceae remain relatively underexplored in China. This study describes the discovery of two novel fungal species, namely *Pleurocordyceps luopingensis***sp. nov**. and *Pseudometarhizium cangyuanense***sp. nov**., during field surveys conducted in Yunnan Province, China. The former belongs to the family Polycephalomycetaceae, and the latter to the family Clavicipitaceae. Both taxa are formally described herein. Detailed morphological descriptions and illustrations are provided, and family-level phylogenies are inferred based on a combined six-locus gene sequence dataset (nr*SSU*, ITS, nr*LSU*, *tef*1-α, *rpb*1, and *rpb*2), thereby confirming the taxonomic placement of the new species. These findings not only enrich the known diversity of entomopathogenic fungi in China but also provide important new evidence for clarifying the phylogenetic relationships among genera and species within Polycephalomycetaceae and Clavicipitaceae, further revealing the unique fungal diversity associated with the special habitats of the Yunnan region.

## Introduction

The order Hypocreales demonstrates the ability to parasitize a wide range of organisms, including plants, insects, nematodes, rotifers, other fungi, and immunocompromised humans (Spatafora et al. 2007; Sung et al. 2007; Kepler et al. 2013, 2017; Lombard et al. 2015; Araújo and Hughes 2016; Wang et al. 2020; Araújo et al. 2022). Within Hypocreales, the families Polycephalomycetaceae and Clavicipitaceae exhibit broad host ranges and diverse ecological roles, parasitizing insects, fungi, and various invertebrates (Spatafora et al. 2007; Sung et al. 2007; Xiao et al. 2023). Notably, Clavicipitaceae displays exceptional host diversity, with known associations involving saprophytic bacteria, symbiotic bacteria, and pathogens linked to soil and plant systems (Kepler et al. 2012a; Zhang et al. 2023). Despite their ecological importance, the taxonomic boundaries and species diversity of these families remain incompletely understood, especially in biodiversity-rich regions.

The family Polycephalomycetaceae was recently established by Xiao et al. (2023) as a phylogenetically distinct lineage sister to Ophiocordycipitaceae. It currently comprises five genera, namely *Polycephalomyces*, *Perennicordyceps*, *Pleurocordyceps*, *Paradingleyomyces*, and *Dingleyomyces* (Xiao et al. 2023; Johnston and Park 2023; Wang et al. 2024). Species of Polycephalomycetaceae display notable ecological versatility, parasitizing both insects and fungi, with such dual parasitic lifestyles being particularly common in the genus *Pleurocordyceps*. *Pleurocordyceps* was established by Wang et al. (2021) based on integrated morphological characteristics and multilocus phylogenetic analyses, with *Pleurocordyceps sinensis* as the type species. Since its establishment, the genus has expanded rapidly and currently includes 24 accepted species (Index Fungorum, accessed 9 February 2026). Species of *Pleurocordyceps* are obligate parasites of insects or fungi and are characterized by fleshy, stipitate stromata with capitate fertile heads, immersed pyriform to ovoid perithecia, cylindrical asci with a distinct apical cap, and filiform ascospores that disarticulate into cylindrical secondary spores. The asexual morph is hyphomycetous and polymorphic, often producing synnemata and two distinct types of phialides (α and β), each giving rise to morphologically different conidia (Matočec et al. 2014; Wang et al. 2021; Xiao et al. 2023).

The family Clavicipitaceae is one of the largest and most diverse families within Hypocreales, currently comprising 58 genera and more than 483 species (Hyde et al. 2024; Wang et al. 2025). Many clavicipitaceous fungi play crucial ecological and applied roles, with several genera – such as *Pochonia*, *Drechmeria*, *Metarhizium*, *Hypocrella*, and *Moelleriella* – being widely used as biocontrol agents against insect pests and plant-parasitic nematodes (Gams and Zare 2003; Chaverri et al. 2008; Spatafora et al. 2015; Mongkolsamrit et al. 2020). The genus *Pseudometarhizium*, established by Chen et al. (2022), is a relatively recent addition to Clavicipitaceae, with *Pseudometarhizium araneogenum* designated as the type species. To date, two species have been described within this genus (Index Fungorum, accessed 9 February 2026). Species of *Pseudometarhizium* are primarily characterized by their asexual morphs, forming light green colonies on PDA, synnematous or mononematous conidiophores, phialides with distinctive helical necks, and unicellular fusiform to ellipsoidal conidia (Chen et al. 2022).

Yunnan Province, located in southwestern China, is recognized as a global biodiversity hotspot and harbors an exceptionally rich diversity of entomopathogenic fungi (Hong Yu 2016). Continued mycological exploration in this region has consistently revealed previously unknown taxa within Hypocreales (Lu et al. 2025). During recent field surveys conducted in Yunnan, many fungal specimens exhibiting distinct morphological features were collected from insect hosts. Integrated morphological examinations and multilocus phylogenetic analyses demonstrated that these specimens represent two previously undescribed species, belonging to *Pleurocordyceps* (Polycephalomycetaceae) and *Pseudometarhizium* (Clavicipitaceae), respectively. In this study, *Pleurocordyceps luopingensis* Hong Yu bis, Z.H Liu, H Wang & X.Y Liu, sp. nov., and *Pseudometarhizium cangyuanense* Hong Yu bis, Z.H Liu, H Wang & X.Y Liu, sp. nov., are described based on detailed morphological characterization and molecular phylogenetic analyses. These findings not only expand the known species diversity of both genera but also contribute to a better understanding of the taxonomy and evolutionary relationships within Polycephalomycetaceae and Clavicipitaceae.

## Materials and methods

### Specimen collection and fungal isolation

Fungal specimens were collected from Yunnan Province, China, with comprehensive documentation of collection site parameters, including altitude, longitude, latitude, and habitat type. Samples were placed in sterile containers, transported to the laboratory, and stored at 4 °C. Specimens were surface-cleaned, assigned unique accession numbers, and air-dried prior to further processing. *Pleurocordyceps* (Polycephalomycetaceae) species isolates were obtained through tissue isolation: sclerotial tissues were surface-sterilized using 75% ethanol, aseptically excised into 2–3 mm segments, and transferred onto potato dextrose agar (PDA) plates. The PDA medium (200 g/L potato extract, 20 g/L dextrose, 20 g/L agar) was supplemented after autoclaving with streptomycin (0.1 g/L) and tetracycline (0.05 g/L) to inhibit bacterial contamination. For *Pseudometarhizium* (Clavicipitaceae), conidia were directly harvested from natural synnemata and inoculated onto PDA. All cultures were incubated at 25 °C. Purified isolates were maintained at 25 °C or preserved on PDA slants at 4 °C. Voucher specimens were deposited in the Yunnan Herbal Herbarium (YHH), and ex-type cultures of both species were deposited in the Yunnan Fungal Culture Collection (YFCC).

### Morphological characterization

To characterize the sexual morphs of *Pleurocordyceps* (Polycephalomycetaceae) species, frozen or hand-cut sections of stromatic fruiting structures were immersed in water and stained with lactophenol cotton blue solution for morphological examination and photomicrography (Wang et al. 2021). Colony morphology was assessed with emphasis on conidial arrangement, phialide structure, and pigment production. To observe the asexual morphs of both species, mycelial fragments from 14-day-old cultures were transferred onto 5 mm agar blocks, which were then mounted on glass slides and incubated in moist chambers to induce sporulation. Morphological features of asexual structures – including conidiophores, phialides, and conidia – were examined and measured using an Olympus BX53 compound microscope (Olympus Corporation, Tokyo, Japan).

### Extraction of DNA, polymerase chain reaction (PCR), and molecular sequencing

Genomic DNA was extracted from the specimens using the Genomic DNA Purification Kit (Qiagen GmbH, Hilden, Germany) according to the manufacturer’s protocol. The purified DNA was used as a template for polymerase chain reaction (PCR) amplification. The nuclear ribosomal small subunit (nr*SSU*) was amplified with the primer pair NS4 (5'-CTTCCGTCAATTCCTTTAAG-3') and NS1 (5'-GTAGTCATATGCTTGTCTC-3') (White et al. 1990). The internal transcribed spacer (ITS) was amplified with the primer pair ITS4 (5'-TCCTCCGCTTATTGATATGC-3') and ITS5 (5'-GGAAGTAAAAGTCGTAACAAGG-3') (White et al. 1990). The nuclear ribosomal large subunit (nr*LSU*) was amplified using primers LR5 (5'-ATCCTGAGGGAAACTTC-3') and LR0R (5'-GTACCCGCTGAACTTAAGC-3') (Vilgalys and Hester 1990; Rehner and Samuels 1994). Amplification of the translation elongation factor 1α (*tef*1-α) was carried out with primers 983F (5'-GCYCCYGGHCAYCGTGAYTTYAT-3') and 2218R (5'-ATGACACCRACRGCRACRGTYTG-3') (Rehner and Buckley 2005). The largest subunit of RNA polymerase II (*rpb*1) was amplified using primers CRPB1A (5'-CAYCCWGGYTTYATCAAGAA-3') and RPB1C (5'-CCNGCDATNTCRTTRTCCATRTA-3') (Castlebury et al. 2004; Bischoff et al. 2006). The second largest subunit of RNA polymerase II (*rpb*2) was amplified with primers fRPB2-5F (5'-GAYGAYMGWGATCAYTTYGG-3') and fRPB2-7cR (5'-CCCATRGCTTGYTTRCCCAT-3') (Liu et al. 2001). PCR amplification and sequencing protocols for these six loci followed the methodology described by Liu et al. (2024a). PCR products were purified using the Gel Extraction and PCR Purification Combo Kit (Beijing Genomics Institute, Shenzhen, China) and sequenced on an automated sequencer (BGI Co., Ltd., Shenzhen, China) using the original amplification primers.

### Phylogenetic analyses

All newly generated sequences were assembled using SeqMan v. 7.1.0 (DNASTAR Inc., Madison, WI, USA). The assembled sequences were deposited at GenBank. The sequences used for phylogenetic analyses are listed in Table 1. The sequences for individual loci were aligned using the MAFFT multiple sequence alignment software v.7.037b (Katoh and Standley 2013) and modified manually using the MEGA software v.6.06 (Tamura et al. 2013). The nr*SSU*, ITS, nr*LSU*, *tef*1-α, *rpb*1, and *rpb*2 sequences were then combined using the “Concatenate Sequence” function in PhyloSuite v.1.2.3 (Zhang et al. 2023). Phylogenetic analyses were carried out using the maximum likelihood (ML) and Bayesian inference (BI) methods. The ML analysis was performed using IQ-TREE v.2.2.0 (Nguyen et al. 2015), whereas the BI analysis was implemented in MrBayes v.3.2.7a (Ronquist et al. 2012). The optimal nucleotide substitution models for both the ML and BI analyses were determined using ModelFinder (Kalyaanamoorthy et al. 2017) under the Akaike information criterion (AIC). The TIM3+R6+F model was identified as the best-fit model for the ML analysis, which was executed with 5,060 ultrafast bootstrap replicates (Minh et al. 2013) in a single run. The GTR+I+G+F model was selected as the optimal model for the BI analysis. For the BI analysis, four independent Markov chain Monte Carlo chains were run for 2 million generations starting from a random tree, with sampling every 100 generations until the average standard deviation of split frequencies dropped below 0.01. The first 25% of sampled trees were discarded as burn-in. The phylogenetic tree was visualized using FigTree v.1.4.3, with branch support values indicated as the maximum likelihood bootstrap proportions (ML-BS) and the Bayesian posterior probabilities (BI-BPP). Final figure annotation and layout refinement were performed in Adobe Illustrator CS6.

**Table 1.** Specimen information and GenBank accession numbers for sequences used in this study.

Taxon	Strain	nr*SSU*	ITS	nr*LSU*	*tef-*1*α*	*rpb*1	*rpb*2	Reference
*Aciculosporium oplismeni*	MAFF246966	-	LC571760	LC571760	LC572040	-	LC572054	Tanaka et al. (2021)
*Aciculosporium take*	MAFF241224	-	LC571753	LC571753	LC572034	-	LC572048	Tanaka et al. (2021)
*Aciculosporium take*	TNS-F-60465	-	LC571755	LC571756	LC572035	-	LC572049	Tanaka et al. (2021)
*Aschersonia badia*	BCC8105	DQ522537	-	DQ518752	DQ522317	DQ522363	DQ522411	Spatafora et al. (2007)
*Aschersonia placenta*	BCC7869	EF469121	-	EF469074	EF469056	EF469085	EF469104	Sung et al. (2007a, b); Kepler et al. (2012b)
*Balansia epichloe*	A.E.G.96-15a	-	-	-	EF468743	-	EF468908	Sung et al. (2007a); Kepler et al. (2012b)
*Claviceps fusiformis*	ATCC26019	DQ522539	JN049817	-	DQ522320	DQ522366	-	Rehner et al. (1995); Spatafora et al. (2007); Kepler et al. (2012b)
*Claviceps purpurea*	GAM12885	-	-	AF543789	AF543778	-	DQ522417	Currie et al. (2003); Spatafora et al. (2007)
*Collarina aurantiaca*	FMR11134	-	KJ807178	KJ807181	-	-	-	Crous et al. (2014)
*Collarina aurantiaca*	FMR11784	-	KJ807177	KJ807180	-	-	-	Crous et al. (2014)
*Conoideocrella luteorostrata*	NHJ11343	-	-	EF468850	EF468801	-	-	Rehner et al. (1995); Spatafora et al. (2007); Kepler et al. (2012b)
*Conoideocrella luteorostrata*	NHJ12516	EF468994	JN049860	EF468849	EF468800	EF468905	EF468946	Sung et al. (2007b)
*Conoideocrella tenuis*	NHJ6293	EU369112	JN049862	EU369044	EU369029	-	EU369087	Johnson et al. (2009); Kepler et al. (2012b)
*Corallocytostroma ornithocopreoides*	WAC8705	-	-	-	LT216546	-	LT216620	Píchová et al. (2018)
*Ephelis japonica*	Eph.oryzae	-	AB038564	-	-	-	-	Tanaka et al. (2001)
*Ephelis tripsaci*	CBS857.72	-	NR153997	NG059240	-	-	-	Hernández-Restrepo et al. (2016)
*Epichloe elymi*	C.Schardl 760	-	-	AY986924	AY986951	DQ000352	-	Chaverri et al. (2005a)
*Epichloe typhina*	ATCC56429		JN049832	-	AF543777	AY489653	DQ522440	Spatafora et al. (2007)
*Heteroepichloe bambusae*	Ba-01	-	AB065426	-	-	-	-	Tanaka et al. (2002)
*Heteroepichloe bambusae*	Bo-01	-	AB065428	-	-	-	-	Tanaka et al. (2002)
*Heteroepichloe sasae*	E.sasae-H	-	AB065432	-	-	-	-	Tanaka et al. (2002)
*Heteroepichloe sasae*	E.sasae-N	-	AB065431	-	-	-	-	Tanaka et al. (2002)
*Keithomyces carneus*	CBS239.32	EF468988	NR_131993	NG057769	EF468789	-	EF468938	Sung et al. (2007a)
*Keithomyces* sp.	CBS126563	MT078871	MT078883	MT078856	-	MT078921	MT078921	Mongkolsamrit et al. (2020)
*Marquandomyces marquandii*	CBS182.27	EF468990	NR_131994	EF468845	EF468793	EF468899	EF468942	Sung et al. (2007b)
*Marquandomyces* sp.	CBS127132	MT078872	MT078882	MT078857	-	-	MT078922	Mongkolsamrit et al. (2020)
*Metapochonia bulbillosa*	CBS145.70	AF339591	MH859529	AF339542	EF468796	-	EF468943	Sung et al. (2001); Sung et al. (2007a); Vu et al. (2019)
*Metapochonia gonioides*	CBS891.72	AF339599	AJ292409	AF339550	DQ522354	-	DQ522458	Zare et al. (2000); Sung et al. (2001); Spatafora et al. (2007)
*Metapochonia sulchlasporia*	CBS251.83	-	NR154139	MH873311	KJ398790	-	KJ398697	Kepler et al. (2014); Vu et al. (2019)
*Metapochonia rubescens*	CBS464.88	AF339615	MH862138	AF339566	EF468797	-	EF468944	Sung et al. (2001); Sung et al. (2007a); Vu et al. (2019)
*Moelleriella phyllogena*	CUP067785	-	-	EU392610	EU392674	-	-	Chaverri et al. (2008)
*Moelleriella phyllogena*	CUP067793	-	-	EU392608	EU392672	-	-	Chaverri et al. (2008)
*Morakotia fusca*	BCC64125	-	-	KY794862	KY794857	-	-	Mongkolsamrit et al. (2021)
*Morakotia fusca*	BCC79272	-	-	KY794861	KY794856	-	-	Mongkolsamrit et al. (2021)
*Metarhizium anisopliae*	CBS130.71	MT078884	MT078884	MT078853	MT078845	MT078861	MT078918	Mongkolsamrit et al. (2020)
*Mycophilomyces periconiae*	CPC27558	-	NR154209	NG059746	-	-	-	Crous et al. (2013)
*Myriogenospora atramentosa*	A.E.G96-32	AY489701	-	AY489733	AY489628	-	DQ522455	Castlebury et al. (2004); Spatafora et al. (2007)
*Neoaraneomyces araneicola*	DY101711	-	MW730520	MW730609	MW753033	-	MW753026	Chen et al. (2022)
*Neoaraneomyces araneicola*	DY101712	-	MW730522	MW730610	MW753034	-	MW753027	Chen et al. (2022)
*Neobarya parasitica*	Marsons/n	-	KP899626	KP899626	-	-	-	Lawrey et al. (2015)
*Nigelia aurantiaca*	BCC13019	GU979939	-	GU979948	GU979957	-	GU979971	Luangsa-ard et al. (2017b)
*Nigelia martiale*	EFCC6863	-	-	JF415974	JF416016	-	-	Kepler et al. (2012b)
*Orbiocrella petchii*	NHJ6209	EU369104	JN049861	EU369039	EU369023	-	EU369081	Johnson et al. (2009); Kepler et al. (2012b)
*Orbiocrella petchii*	NHJ6240	EU369103	-	EU369038	EU369022	-	EU369082	Johnson et al. (2009)
*Purpureomyces maesotensis*	BCC88441	-	MN781916	MN781877	MN781734	-	MN781824	Mongkolsamrit et al. (2020)
*Regiocrella camerunensis*	ARSEF7682	-	-	DQ118735	DQ118743	DQ127234	-	Chaverri et al. (2005b)
*Rotiferophthora angustispora*	CBS101437	AF339584	AJ292412	AF339535	AF543776	DQ522402	DQ522460	Zare et al. (2000); Sung et al. (2001); Currie et al. (2003); Spatafora et al. (2007)
*Samuelsia chalalensis*	CUP067856	-	-	EU392637	EU392691	EU392743	-	Chaverri et al. (2008)
*Samuelsia mundiveteris*	BCC40021	-	-	GU552152	GU552145	-	-	Mongkolsamrit et al. (2017)
*Samuelsia rufobrunnea*	CUP067858	-	-	AY986918	AY986944	DQ000345	-	Chaverri et al. (2005a)
*Shimizuomyces paradoxus*	EFCC6279	EF469131	JN049847	EF469084	EF469071	EF469100	EF469117	Sung et al. (2007a, b); Kepler et al. (2012b)
*Shimizuomyces paradoxus*	EFCC6564	EF469130	-	EF469083	EF469072	-	EF469118	Sung et al. (2007ab)
*Sungia yongmunensis*	EFCC2131	EF468977	JN049856	EF468833	EF468770	-	-	Sung et al. (2007a); Kepler et al. (2012b)
*Sungia yongmunensis*	EFCC2135	EF468979	-	EF468834	EF468769	-	-	Sung et al. (2007a)
*Tyrannicordyceps fratricida*	TNS19011	-	JQ349068	JQ257023	JQ257028	-	JQ257021	Kepler et al. (2012a)
*Ustilaginoidea virens*	ATCC16180	-	-	-	JQ257026	-	JQ257019	Kepler et al. (2012a)
*Ustilaginoidea virens*	MAFF240421	-	JQ349068	JQ257011	JQ257024	-	JQ257017	Kepler et al. (2012a)
*Yosiokobayasia kusanagiensis*	TNS-F18494	JF415954	JN049873	JF415972	JF416014	-	-	Kepler et al. (2012a)
*Periglandula ipomoeae*	IasaF13	-	-	-	KP689568	-	KP689517	Steiner et al. (2011)
*Papiliomyces liangshanensis*	EFCC1452	EF468962	-	EF468815	EF468756	-	-	Sung et al. (2007a)
*Papiliomyces liangshanensis*	EFCC1523	EF468961	-	EF468814	EF468755	-	EF468918	Sung et al. (2007a)
*Papiliomyces shibinensis*	GZUHSB13050311	KR153588	NR154178	-	KR153589	-	-	Wen et al. (2015)
*Parametarhizium hingganense*	CGMCC 19144	MN055706	MN055703	MN061635	MN065770	-	MT939494	Gao et al. (2021)
*Paraneoaraneomyces sinensis*	ZY22.006	OQ709248	OQ709254	OQ709260	OQ719626	-	OQ719621	Zhang et al. (2023)
*Paraneoaraneomyces sinensis*	ZY22.007	OQ709249	OQ709255	OQ709261	OQ719627	-	OQ719622	Zhang et al. (2023)
*Paraneoaraneomyces sinensis*	ZY22.008	OQ709250	OQ709256	OQ709262	OQ719628	-	OQ719623	Zhang et al. (2023)
*Pseudometarhizium araneogenum*	DY101801^T^	-	MW730536	MW730623	MW753039	-	MW753032	Chen et al. (2022)
*Pseudometarhizium araneogenum*	DY101802	-	MW730545	MW730625	MW753040	-	-	Chen et al. (2022)
*Pseudometarhizium lepidopterorum*	SD05361^T^	-	MW730543	MW730624	MW753041	-	-	Chen et al. (2022)
*Pseudometarhizium lepidopterorum*	SD05362	-	MW730611	MW730629	MW753042	-	-	Chen et al. (2022)
*Pseudometarhizium cangyuanense*	YFCC08069631^T^	-	-	PX901754	-	PZ280815	PZ269979	**This study**
*Parepichloe cinerea*	Ne-01	-	AB065425	-	-	-	-	Tanaka et al. (2002)
*Pochonia boninensis*	JCM18597	AB758255	AB709858	AB709831	AB758463	-	AB758693	Nonaka et al. (2013)
*Pochonia globispora*	CBS203.86	-	DQ516079	-	-	-	-	Vu et al. (2019)
*Pleurocordyceps agarica*	YHHPA1305^T^	KP276655	KP276651	-	KP276659	KP276663	KP276667	Wang et al. (2015b)
*Pleurocordyceps agarica*	YHCPA1307	KP276658	KP276654	-	KP276662	KP276666	KP276670	Wang et al. (2015b)
*Pleurocordyceps aurantiacus*	MFLUCC17-2113^T^	MG136904	MG136916	MG136910	MG136875	MG136866	MG136870	Xiao et al. (2019)
*Pleurocordyceps aurantiacus*	MFLU17-1394	MG136906	MG136918	MG136912	MG136876	MG136867	MG136872	Xiao et al. (2019)
*Pleurocordyceps clavisynnema*	GZLG23-102	-	OQ968788	OQ968796	OQ982009	-	-	Xiao et al. (2024)
*Pleurocordyceps clavisynnema*	GZCC22-2042	OQ968805	OQ968789	OQ968797	OQ982008	OQ981998	OQ982004	Xiao et al. (2024)
*Pleurocordyceps formosus*	ARSEF1424	KF049615	KF049661	KF049634	KF049689	KF049651	KF049671	Kepler et al. (2013)
*Pleurocordyceps formosus*	MFLU18-0162	MK863043	MK863250	MK863050	MK860188	-	-	Unpublished
*Pleurocordyceps heilongtanensis*	KUMCCC3008	OQ172111	OQ172091	OQ172063	OQ459731	OQ459759	OQ459805	Xiao et al. (2023)
*Pleurocordyceps kanzashianus*	-	AB027325	AB027371	-	-	-	-	Nikoh et al. (2000)
*Pleurocordyceps lanceolatus*	GACPCC17-2005^T^	OQ172109	-	OQ172047	OQ459727	OQ459755	OQ459801	Xiao et al. (2023)
*Pleurocordyceps lanceolatus*	GACP17-2004^T^	OQ172110	OQ172076	OQ172046	OQ459726	OQ459754	OQ459800	Xiao et al. (2023)
*Pleurocordyceps lianzhouensis*	HIMGD20918	KF226245	EU149921	KF226246	KF226248	KF226247	-	Zhang et al. (2007)
*Pleurocordyceps lianzhouensis*	GIMYY9603	KF226249	EU149922	KF226250	KF226252	KF226251	-	Zhang et al. (2007)
*Pleurocordyceps litangensis*	YFCC06109293^T^	PP541902	PP410597	PP410593	PP550103	PP697751	PP550107	Liu et al. (2024a)
*Pleurocordyceps litangensis*	YFCC06109295	PP541905	PP410600	PP410596	PP550104	PP697754	PP550108	Liu et al. (2024a)
*Pleurocordyceps marginaliradians*	MFLU17-1582	MG136908	MG136920	MG136914	MG136878	MG136869	MG271931	Xiao et al. (2019)
*Pleurocordyceps multisynnema*	GZLG23-101	OQ968802	OQ968792	OQ968800	-	-	OQ982002	Xiao et al. (2024)
*Pleurocordyceps multisynnema*	GZCC22-2041	OQ968803	OQ968793	OQ968801	OQ982010	OQ981997	OQ982003	Xiao et al. (2024)
*Pleurocordyceps neoagarica*	GZLG23-103	-	OQ968790	OQ968795	-	-	-	Xiao et al. (2024)
*Pleurocordyceps neoagarica*	GZCC22-2043	OQ968804	OQ968791	OQ968794	OQ982007	OQ981996	OQ981999	Xiao et al. (2024)
*Pleurocordyceps nipponica*	NHJ4268	KF049621	KF049657	KF049639	MF416517	MF416676	KF049676	Kepler et al. (2013)
*Pleurocordyceps nipponica*	BCC1682	KF049620	KF049664	KF049638	KF049694	-	-	Kepler et al. (2013)
*Pleurocordyceps nipponica*	NBRC101408	JN941751	JN943303	JN941390	-	JN992485	-	Schoch et al. (2012)
*Pleurocordyceps nipponica*	BCC2325	KF049622	KF049665	KF049640	KF049696	KF049655	KF049677	Kepler et al. (2013)
*Pleurocordyceps nutansis*	GACP19-1906	OQ172117	OQ172079	OQ172049	OQ459737	OQ459763	OQ459809	Xiao et al. (2023)
*Pleurocordyceps nutansis*	MFLU21-0275^T^	OQ172119	OQ172073	OQ172048	OQ459739	OQ459765	OQ459811	Xiao et al. (2023)
*Pleurocordyceps onorei*	BRACR23902	-	KU898841	-	-	-	-	Crous et al. (2017)
*Pleurocordyceps onorei*	BRACR23904	-	KU898843	-	-	-	-	Crous et al. (2017)
*Pleurocordyceps phaothaiensis*	BCC84557	-	MF959734	MF959738	MF959741	MF959746	-	Wang et al. (2021)
*Pleurocordyceps phaothaiensis*	BCC84553^T^	-	MF959733	MF959737	MF959742	MF959745	-	Wang et al. (2021)
*Pleurocordyceps puerensis*	GACPY117	PP707759	-	PP616709	PP654214	PP654216		Cao et al. (2025)
*Pleurocordyceps ramosopulvinatus*	EFCC5566	-	KF049658	KF049627	KF049682	KF049645	-	Kepler et al. (2013)
*Pleurocordyceps ramosopulvinatus*	SU65	-	-	DQ118742	DQ118753	DQ127244	-	Chaverri et al. (2005a)
*Pleurocordyceps ramosopulvinatus*	-	AB027326	AB027372	-	-	-	-	Nikoh and Fukatsu (2000)
*Pleurocordyceps ramosus like*	NBRC101760	MN586818	MN586827	MN586836	MN598051	MN598042	MN598060	Wang et al. (2020)
*Pleurocordyceps ramosus like*	NBRC109984	MN586819	MN586828	MN586837	MN598052	MN598043	-	Wang et al. (2020)
*Pleurocordyceps sanduensis*	GZLG23-104	-	OQ968786	OQ968798	OQ982005	-	OQ982000	Xiao et al. (2024)
*Pleurocordyceps sanduensis*	GZCC22-2044	OQ968806	OQ968787	OQ968799	OQ982006	OQ981995	OQ982001	Xiao et al. (2024)
*Pleurocordyceps sinensis*	CGMCC3.19069	MH454346	MH459160	-	-	-	-	Sun et al. (2019)
*Pleurocordyceps sinensis*	CN80-2^T^	HQ832887	HQ832884	HQ832886	HQ832890	HQ832888	HQ832889	Wang et al. (2012)
*Pleurocordyceps sinensis*	HMAS43720^T^	-	NR119928	NG042573	-	-	-	Wang et al. (2012)
*Pleurocordyceps* sp.	BCC2637	KF049619	KF049663	KF049637	KF049693	-	KF049675	Kepler et al. (2013)
*Pleurocordyceps* sp.	JB07081608	KF049616	-	KF049635	KF049690	KF049652	KF049672	Kepler et al. (2013)
*Pleurocordyceps* sp.	JB070817 07b	KF049617	-	-	KF049691	KF049653	KF049673	Kepler et al. (2013)
*Pleurocordyceps* sp.	NBRC109987	-	-	AB925983	-	-	-	Unpublished
*Pleurocordyceps* sp.	NBRC109988	-	-	AB925984	-	-	-	Unpublished
*Pleurocordyceps* sp.	NBRC109990	-	-	AB925968	-	-	-	Unpublished
*Pleurocordyceps* sp.	NBRC110224	-	-	AB925969	-	-	-	Unpublished
*Pleurocordyceps* sp.	GIMCC3570	JX006097	-	JX006098	JX006100	JX006101	-	Zhong et al. (2016)
*Pleurocordyceps tomentosus*	BL4	KF049623	KF049666	KF049641	KF049697	KF049656	KF049678	Kepler et al. (2013)
*Pleurocordyceps vitellina*	KUMCCC3005	-	OQ172088	OQ172060	OQ459728	OQ459756	OQ459802	Xiao et al. (2023)
*Pleurocordyceps vitellina*	KUMCCC3006	-	OQ172089	OQ172061	OQ459729	OQ459757	OQ459803	Xiao et al. (2023)
*Pleurocordyceps vitellina*	KUMCCC3007	-	OQ172090	OQ172062	OQ459730	OQ459758	OQ459804	Xiao et al. (2023)
*Pleurocordyceps yunnanensis*	YHCPY1005	KF977848	KF977848	KF977848	KF977850	KF977852	KF977854	Wang et al. (2015a)
*Pleurocordyceps yunnanensis*	YHHPY1006^T^	KF977849	KF977849	KF977849	KF977851	KF977853	KF977855	Wang et al. (2015a)
*Pleurocordyceps luopingensis*	YFCC07089629^T^	PX901752	PX901750	PX901755	PZ279702	PZ279704	-	**This study**
*Pleurocordyceps luopingensis*	YFCC07089630	PX901753	PX901751	PX901756	PZ279703	PZ279705	-	**This study**
*Dingleyomyces lloydii*	PDD1212154^T^	OR647563	OR602634	OR602640	OR588853	OR588860	OR588858	Johnston and Park (2023)
*Paradingleyomyces lepidopterorum*	HKAS131926^T^	-	OR878363	OR828238	-	OR829674	OR880683	Wang et al. (2024)
*Paradingleyomyces lepidopterorum*	HKAS131927	-	OR878364	OR828239	OR880679	OR829675	-	Wang et al. (2024)
*Paradingleyomyces lepidopterorum*	HKAS131921	-	-	OR828242	-	OR829678	-	Wang et al. (2024)
*Perennicordyceps cuboidea*	NBRC103836	JN941721	JN943332	JN941420	AB972951	JN992455	AB972955	Schoch et al. (2012)
*Perennicordyceps cuboidea*	NBRC101740	JN941724	JN943331	JN941417	KF049684	JN992458	-	Schoch et al. (2012)
*Perennicordyceps ryogamiensis*	NBRC103842	JN941701	JN943345	JN941440	-	JN992435	-	Schoch et al. (2012)
*Perennicordyceps ryogamiensis*	NBRC101751	JN941703	JN943343	JN941438	KF049688	JN992437	-	Schoch et al. (2012)
*Polycephalomyces albiramus*	GACP21-XS08^T^	OQ172115	OQ172092	OQ172037	OQ459735	OQ459761	OQ459807	Xiao et al. (2023)
*Polycephalomyces albiramus*	GACPCC21-XS08^T^	OQ172116	OQ172093	OQ172038	OQ459736	OQ459762	OQ459808	Xiao et al. (2023)
*Polycephalomyces formosus*	NBRC109993^T^	MN586824	MN586833	MN586842	MN598057	MN598048	MN598064	Wang et al. (2021)
*Polycephalomyces formosus*	NBRC109994	MN586825	MN586834	MN586843	MN598058	MN598049	MN598065	Wang et al. (2021)
*Cordyceps militaris*	OSC93623	AY184977	JN049825	AY184966	DQ522332	DQ522377	AY545732	Kepler et al. (2012)
*Cordyceps militaris*	YFCC6587	MN576762	-	MN576818	MN576988	MN576878	MN576932	Wang et al. (2020)

^T^ represents type strain or type specimen.

## Results

### Phylogenetic analyses

**Figure 1.**
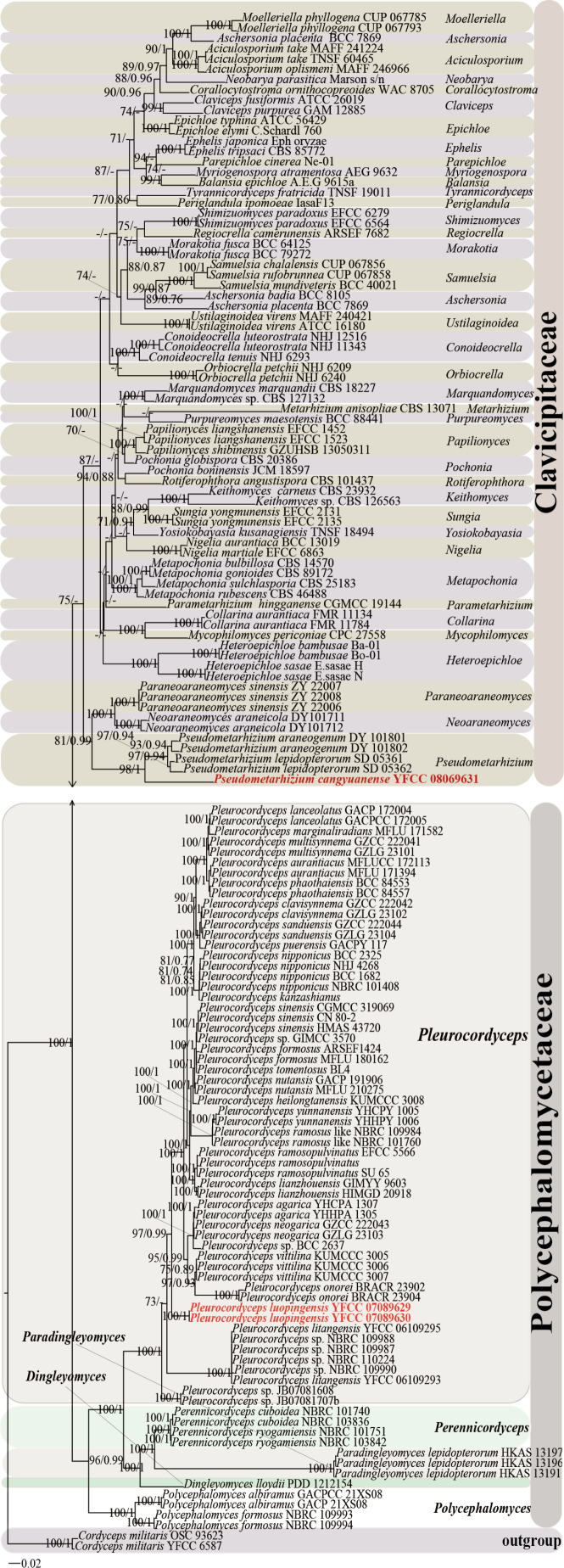
Molecular phylogenetic analyses were conducted using the ML and BI methods based on concatenated sequence data from the nr*SSU*, ITS, nr*LSU*, *tef*1-α, *rpb*1, and *rpb*2 loci. Two strains of *Cordyceps militaris* (OSC93623 and YFCC 6587) were designated as outgroup taxa. Statistical support values—specifically the ML bootstrap support (BP ≥ 70%) and the BI posterior probabilities (PP ≥ 0.70)—are indicated at the corresponding nodes. Isolates highlighted in red represent those newly included in this study.

Phylogenetic analysis of *Pleurocordyceps* and *Pseudometarhizium* species was conducted using a data matrix comprising sequences from 146 samples (Table 1). Two representative strains of *C. militaris* (OSC 93623 and YFCC 6587) were designated as the outgroup. The final aligned dataset spanned 5,981 base pairs (bp) and was partitioned as follows: nr*SSU* (1,022 bp), ITS (849 bp), nr*LSU* (920 bp), *tef*1-α (992 bp), *rpb*1 (1,072 bp), and *rpb*2 (1,126 bp). Both the BI and ML analyses yielded phylogenetic trees with congruent topologies, resolving most *Pleurocordyceps* and *Pseudometarhizium* lineages into well-defined clades (Fig. 1). The overall tree topologies were consistent with those reported in previous studies. The two newly identified species were phylogenetically placed within strongly supported clades corresponding to their respective genera: *Pl. luopingensis* formed a distinct and independent lineage within the genus *Pleurocordyceps*, while *Pseudometarhizium cangyuanense* clustered with *Ps. araneogenum* and *Ps. lepidopterorum*. Each novel species occupied a separate, well-resolved branch within its clade, clearly distinguishing it from its closest relatives (Fig. 1).

**Figure 2.**
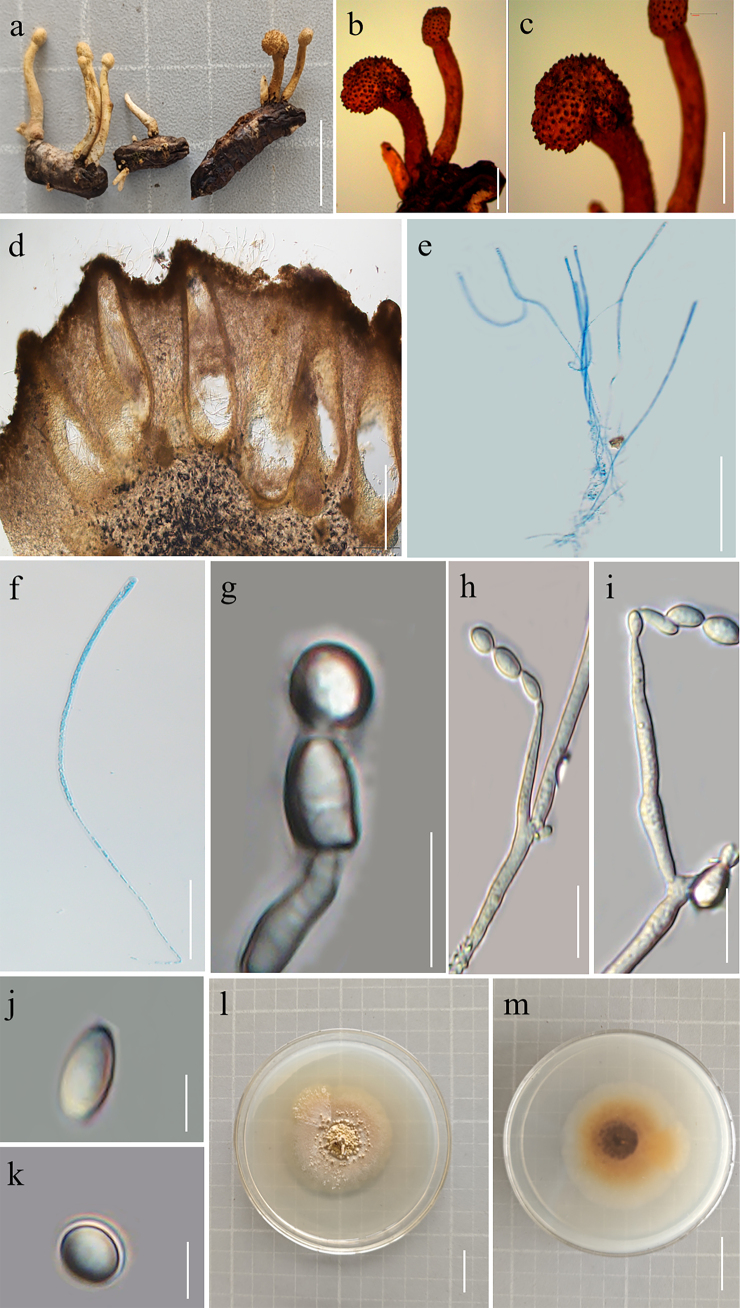
Morphological characteristics of *Pleurocordyceps luopingensis* (Type YHH-PLU-20507130; type living culture, YFCC 07089629). **a**. Stromata of *Pleurocordyceps luopingensis* emerging from the host; **b, c**. Fertile heads; **d**. Peridium; **e, f**. Asci; **g**. α-phialides; **h, i**. β-phialides; **j**. β-conidia; **k**. α-conidia; **l, m**. Colonies on PDA after 54 days (**l**. Obverse; **m**. Reverse). Scale bars: 1 cm (**a**); 2 cm (**b, c, l, m**); 400 µm (**d**); 100 µm (**e**); 50 µm (**f**); 10 µm (**g, h, i**); 5 µm (**j, k**).

**Figure 3.**
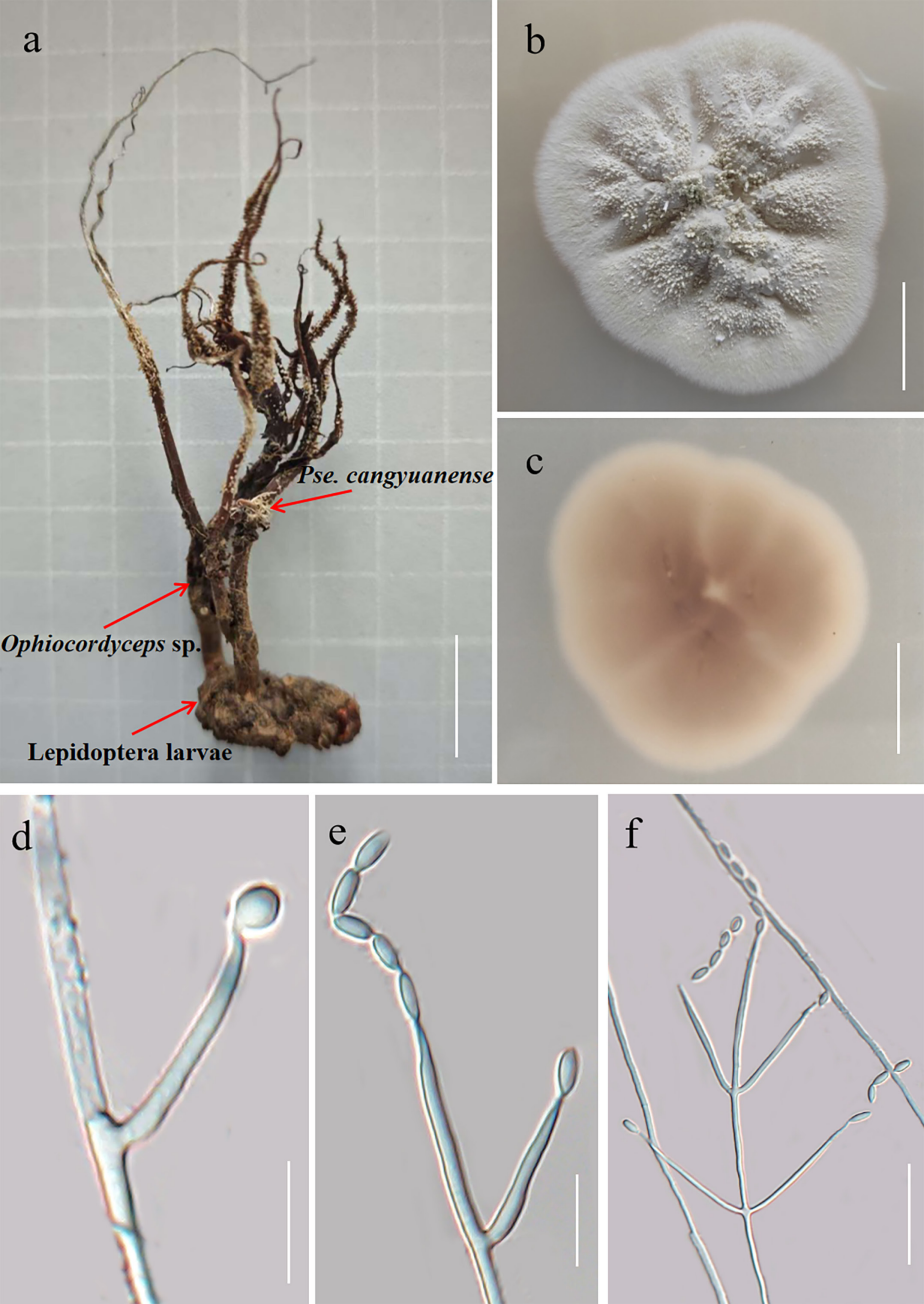
Morphology of *Pseudometarhizium cangyuanense* (Type YHH-MCA-20508133; type living culture, YFCC 08069631). **a**. Stromata of *Pseudometarhizium cangyuanense* growing from the host; **b, c**. Colonies on PDA after 14 days (**b**. Obverse; **c**. Reverse); **d–f**. Phialides and conidia. Scale bars: 2 cm (**a**); 1 cm (**b, c**); 10 µm (**d, e**); 10 µm (**f**).

### Taxonomy

#### Pleurocordyceps luopingensis

Taxon classificationFungiSordariomycetesPolycephalomycetaceae

Hong Yu bis, Z.H Liu, H Wang & X.Y Liu sp. nov.

3A64AE15-C69B-57F2-AB2A-337D1AE89689

862005

Fig. 2

##### Etymology.

Named after Luoping County, in reference to the locality where the species was first collected.

##### Type.

Specimens were collected from Hemiptera larvae on 13 July 2025 at an altitude of 1545.5 m, with geographic coordinates 25°15'24"N, 104°42'12"E; by Hong Yu; designated as holotype specimen YHH-PLU-20507130 and type living culture, YFCC 07089629.

##### *Sexual morph*.

Parasitic on adults of Hemiptera (Fig. 2). Host bodies range from 10–23 mm in diameter, exhibiting segmentation, brown to dark brown pigmentation, and a hard, rugose surface. Stromata are cylindrical, 2.1–15.2 × 0.5–2.1 mm (x̄ = 4.5 × 1.2 mm, n = 20), light yellow to pale yellow in color, and occur either abundantly or singly. Stipes are 1.3–5.6 × 0.5–2.7 mm (x̄ = 3.4 × 1.4 mm, n = 20), light yellow, disc-shaped to cylindrical, with some terminating in fertile cushions. Fertile cushions are pale yellow to yellow, tapering, 0.2–0.6 × 0.1–0.2 mm (x̄ = 0.4 × 0.5 mm, n = 30). Perithecia are immersed, densely packed, obpyriform, ostiolate, and measure 150–520 × 90–325 μm (x̄ = 360 × 120 μm, n = 30). Asci are cylindrical, 230–746 × 1.1–2.7 μm (x̄ = 420 × 1.2 μm, n = 30), with a thickened apex bearing a hyaline apical cap 1.1–2.2 × 2.8–3.6 μm (x̄ = 1.5 × 3.2 μm, n = 30) in size.

***Asexual morph***. Colonies on PDA reach a diameter of 4.1–4.9 cm after 54 days of incubation at 25 °C, indicating slow growth. Colonies are off-white to gray in color and leathery in texture, with multiple granular protrusions on the surface; the reverse side is grayish yellow. Synnemata emerge from the colony center in annular arrangements, measuring 0.5–4 cm in length and 0.2–0.5 mm in width. These structures are khaki-colored, erect or flexuous, and apically umbrella-shaped. A pale yellow conidial mass accumulates at the mid-region or apex of the synnemata. Two types of phialides are observed. α-Phialides are hyaline, smooth-walled, subulate, and clustered, measuring 6.2–13.5 × 1.3–2.4 µm (x̄ = 7.2 × 1.5 μm, n = 30). α-conidia are unicellular, hyaline, smooth-walled, and oval, measuring 1.1–2.1 × 0.9–1.6 (x̄ = 1.3 × 1.2 μm, n = 30) µm. β-phialides measure 9.5–17.7 × 0.7–2.1 µm (x̄ = 10.2 × 1.1 μm, n = 30), arise directly from hyphae, and are solitary, lanceolate, hyaline, and smooth-walled. β-conidia are fusiform, hyaline, aseptate, smooth-walled, asymmetrical, and arranged in chains, measuring 3.3–5.6 × 2.1–3.4 µm (x̄ = 3.5 × 2.9 μm, n = 30).

##### Host.

Hemiptera adults.

##### Known distribution.

Yunnan Province, China.

##### Additional specimens examined.

Specimens were collected from Hemiptera larvae on 13 July 2025 at an altitude of 1545.5 m, with geographic coordinates 25°15'24"N, 104°42'12"E; by Hong Yu and Zuoheng Liu; designated as ex-holotype specimen YHH-PLU-20507131 and ex-holotype living culture YFCC 07089630.

##### Commentary.

Phylogenetic analysis based on the combined dataset showed that *Pleurocordyceps luopingensis* formed a distinct and fully supported lineage (BS/BPP = 100/1.00) within the genus *Pleurocordyceps* (Fig. 1). In contrast to other species within the genus *Pleurocordyceps*, *Pl. luopingensis* and *Pl. yunnanensis* share the same host, the Hemiptera adults (Tables 2, 3). *Pl. luopingensis* is distinct from the species *Pl. yunnanensis* in that it produces stromata that are light yellow to pale yellow, colonies that are off-white to gray and leathery in texture, and α-conidia that are oval (Tables 2, 3). Thus, *Pl. luopingensis* is introduced as a new species under *Pleurocordyceps*.

**Table 2.** Morphological comparison of sexual morph species of the genus *Pleurocordyceps*.

Species	Host	Stromata	Stipe	Fertile part	Perithecia	Asci	Ascospores	Part-spores	Reference
*Pleurocordyceps marginaliradians*	Cossidae larvae	mostly single, stipitate, unbranched or branched brown to yellow	Cylindrical, brown to yellow, with one or two fertile head	lateral, globose to subglobose, pale yellow to yellow, with protruding ostiolar neck	immersed, yellow, flask shaped	hyaline, filiform	As long as the asci, easily breaking into part-spores, filiform	cylindrical, straight	Xiao et al. (2018)
*Pleurocordyceps kanzashianus*	Cicadidae nymph	Stipitate, branched, polycephalous	Cylindrical, leather, rough, terminal branched	Apical or either Side spherical, yellow, small	Entirely immersed, flask-shaped		Break into part-spores	both truncate	Kobayasi and Shimizu (1983)
*Pleurocordyceps lianzhouensis*	Lepidoptera larva or *Ophiocordyceps crinalis*	Numerous, simple, fleshy, arising from the head, abdomen, and back of the host	Cylindrical, reddish brown or cinnamon colored	Hemispherical or capiform, pale yellow or pale yellowish brown, acrogenous	Narrowly ovoid with protruding apices, vertically	Asci: cylindrical, caps: hemi spherical	Filiform, break into part spores	Cylindrical	Wang et al. (2014)
*Pleurocordyceps nipponica*	Cicadidae	Solitary or arranged in twos or threes in height, often highly branched and polycephalous	Cylindrical sim plex, irregularly branched sometimes base has two parts	Terminal or lateral, depressed, fleshy, sometimes crowded	Immersed flask-shaped or ovoid		Filiform, disarticulating into part-spores	truncate	Kobayasi (1939)
*Pleurocordyceps onorei*	Caterpillar (Arctiinae)	Numerous, soli tary, simple or 2–3 times branched, ampulliform, thickened at the base, cinnamon brown, darker when wet, fading with age and drying to greyish brown	Cylindrical	Subapical, forming lateral pads around stipe, pale brown to ochraceous orange, with sterile apical part	Pyri form, with dark brown protruding apices		Filiform, cylindrical, breaking to part-spores	truncate, bacilliform	Crous et al. (2017)
*Pleurocordyceps parvicapitata*	*Elaphomyces* sp.	branched, brown to yellow, hard, cylindrical	brown to dark brown, branched, with small capitate head	yellow to pale yellow, single or capitate, rough, with ostiole	immersed, crowded, ovoid to obpyriform, yellow	filiform	filiform, hyaline, multiseptated	oblong to cylindrical, one-celled	Xiao et al. (2023)
*Pleurocordyceps ramosopulvinatus*	Cicada nymph		Cylindrical, leathery, apically branching, glabrous, pallid ocherous in color	Fertile area cushion shaped to globose, with aggregated perithecia forming composite heads	Pyriform,	with apical cap diameter	Filiform, disarticulating into part-spores	3×1 truncate	Kobayasi and Shimizu (1983)
*Pleurocordyceps luopingensis*	Hemiptera adults		Stromata are cylindrical, light yellow to pale yellow in color, and occur either abundantly or singly.	Stipes light yellow, disc-shaped to cylindrical, with some terminating in fertile cushions. Fertile cushions are pale yellow to yellow.	Perithecia are immersed, densely packed, obpyriform, ostiolate.	Asci are cylindrical, with a thickened apex bearing a hyaline apical cap			**This study**

**Table 3.** Morphological comparison of asexual morph species of *Pleurocordyceps*.

Species	Host	Synnemata	Phialides	Conidia	References
*Pleurocordyceps agarica*	*Ophiocordyceps* sp. or melolonthid larvae	Solitary, unbranched, agaricshaped; conidial mass pileus-like, light yellow to pale brown	α-phialides lanceolate; β-phialides narrowly lageniform or subulate	α-conidia globose to subglobose; β-conidia fusiform, catenate or clump together	Wang et al. (2015b)
*Pleurocordyceps aurantiacus*	Coleoptera larvae or *Ophiocordyceps barnesii*	Emerging after 30 days, solitary or not solitary, branched or unbranched, showing 1–2 radiating ring-like distributions	α-phialides, narrowly lageniform. β-phialides, lanceolate or narrowly lageniform	α-conidia, globose to subglobose. β-conidia, fusiform	Xiao et al. (2018)
*Pleurocordyceps lanceolatus*	Lepidoptera larvae	lanceolate to corniform, solitary to crowded, stipitate, usually unbranched, rarely branched on the PDA, yellow to yellowish on the fresh specimen, covered with conidial masses, white on the PDA	α-phialides directly from hyphae, solitary, usually unbranched, subulate at the base, tapering into a long neck; β-phialides branched into two or three phialides, narrowly lageniform to lanceolate	α-conidia spherical, forming slimy conidial masses along the synnemata; β-conidia fusiform	Xiao et al. (2023)
*Pleurocordyceps marginaliradians*	Cossidae larva	Emerging after 14 days, single or branched into two or three branched, showing 1–2 radiating ring-like distributions	α-phialides, elongate lageniform; β-phialides, narrow slender to narrow lageniform	α-conidia globose, catenate, one-celled, pale yellow slimy in mass. β-conidia fusiform, one-celled	Xiao et al. (2018)
*Pleurocordyceps parvicapitata*	*Perennicordyceps elaphomyceticola*	Absent	Phialides, cylindrical at the base, tapering into a long neck	globose to subglobose	Xiao et al. (2023)
*Pleurocordyceps sinensis*	Lepidoptera larvae or *Ophiocordyceps sinensis*	Solitary, crowded, branched or unbranched, conidial mass yellow or yellow-orange	Lanceolate or narrowly lageni-form	α-conidia, ovoid; β-conidia, fusiform	Chen et al. (1984); Wang et al. (2012)
*Pleurocordyceps vitellina*	*Ophiocordyceps nigrella*	Absent	α-phialides, hyaline, smooth, elongated lageniform, crowed, gathered in the middle of colony. β-phialides, hyaline, smooth, directly growing from hyphae, with or without metula at the base, solitary, lanceolate, ovate at the base, tapering into a short neck	α-conidia spherical, one-celled, smooth-walled. β-conidia fusiform, catenulate	Xiao et al. (2023)
*Pleurocordyceps yunnanensis*	Hemiptera adults or *Ophiocordyceps nutans*	Solitary, caespitose or crowded, branched or unbranched; conidial mass white to yellow–brown	α-phialides cylindrical to subulate; β-phialides narrowly lageniform or subulate	α-conidia subglobose, ellipsoidal; β-conidia fusiform, catenate or clump together	Wang et al. (2015a)
*Pleurocordyceps nutansis*	*Ophiocordyceps nutans*	cylindrical, clavate, capitate, stipitate, crowded, simple, white to yellowish	Two types, both of the types observed on the same synnema. α-phialides, gathered at the apex of the synnema, arranged in a parallel palisade-like layer around the apex of the fertile head, hyaline, usually branched into 2–6 phialides, narrowly slender lanceolate; β-phialides, solitary, scattered along the stipe, lanceolate, ovate at the base, tapering into a long neck	α-conidia, spherical, forming slimy conidial masses on the fertile head; β-conidia fusiform, produced along stipe of the synnema	Xiao et al. (2023)
*Pleurocordyceps heilongtanensis*	*Ophiocordyceps* sp.	scattered on the surface of host, cylindrical, stipitate, unbranched, white, with or without fertile head	α-phialides, hyaline, smooth, elongated lageniform, caespitose, palisade-like, crowed, gathered in the top of synnema, mostly branched into 2–4 phialides. β-phialides hyaline, smooth, solitary, branched into two or three phialides, with or without metula at the base, directly growing from hyphae	α-conidia, subglobose to ovoid, in yellowish slimy mass. β-conidia fusiform, one-celled	Xiao et al. (2023)
*Pleurocordyceps lianzhouensis*	Lepidoptera larva or *Ophiocordyceps crinalis*	Unbranched or dichotomously branched, conidial mass not seen	In whorls or intercalary and terminal, terminally awl-shaped	Ellipsoidal, oblong to cylindrical	Wang et al. (2014)
*Pleurocordyceps litangensis*	*Ophiocordyceps sinensis*	Absent	α-phialides acropleurogenous solitary on hyphae; spear point. β-phialides terminal on solitary on hyphae; subulate	α-conidia ovoid or ellipt; β-conidia fusiform,	Liu et al. (2024a)
*Pleurocordyceps fusiformispora*	Lepidoptera larva or *Ophiocordyceps* sp.	Flaky, branched, showing radiating distributions.	α-phialides narrow lageniform or subulate. β-phialides solitary on hyphae, lanceolate.	α-conidia ovoid or elliptic; β-conidia fusiform or long fusiform.	Liu et al. (2024b)
*Pleurocordyceps clavisynnema*	*Ophiocordyceps neogryllotalpae*	clavate or with a mucronate apex, solitary, unbranched	α-, β-phialides smooth, hyaline, solitary.	α-conidia globose, one-celled, smooth-walled; β-conidia hyaline, fusiform, one-celled, smooth	Xiao et al. (2024)
*Pleurocordyceps multisynnema*	*Paraisaria* sp.	single, light yellow, cylindrical, without a fertile head, stipitate	α-phialides solitary, narrow lanceolate, from the synnema; β-phialides, directly from hyphae, solitary, narrow lanceolate, suddenly tapering from the bottom to the apex.	α-conidia, spherical, one-celled, smooth. β-conidia, fusiform, one-celled, smooth.	Xiao et al. (2024)
*Pleurocordyceps neoagarica*	*Ophiocordyceps neogryllotalpae*	solitary, non-branched, displaying several ring-like distributions.	Phialides, one type, narrowly slim lanceolate, cylindrical at the base, tapered into a long neck, hyaline, smooth.	Conidia, arising from the apex of phialides, globose, one-celled, hyaline.	Xiao et al. (2024)
*Pleurocordyceps sanduensis*	*Ophiocordyceps neogryllotalpae*	solitary, unbranched, distribution at the edge, with small or without a fertile head.	α-, β-phialides, smooth, hyaline, solitary.	α-conidia, globose, unicellular, smooth-walled; β-conidia fusiform, unicellular, hyaline, smooth-walled.	Xiao et al. (2024)
*Pleurocordyceps longiphialis*	Scarabaeoidea larva	Stromata arise from the head and thoracic region of the host, solitary or paired, simple or branched, clavate, yellow to brown	Phialides have two types, α-phialides solitary, cylindrical or lanceolate. β-phialides terminal on solitary on hyphae; lageniform or subulate.	α-conidia one-celled, hyaline, smooth, subglobose, ovoid to ellipsoidal. β-conidia one-celled, hyaline, smooth, fusiform, oblong-elliptical to ellipsoidal, solitary or aggregated in long chains.	Dong et al. (2026)
*Pleurocordyceps luopingensis*	Hemiptera adults	Synnemata emerge from the colony center in annular arrangements; structures are khaki-colored, erect or flexuous, and apically umbrella-shaped.	Two types of phialides are observed. α-Phialides are hyaline, smooth-walled, subulate, and clustered. β-phialides arise directly from hyphae and are solitary, lanceolate, hyaline, and smooth-walled.	α-conidia are unicellular, hyaline, smooth-walled, and oval. β-conidia are fusiform, hyaline, aseptate, smooth-walled, asymmetrical, and arranged in chains.	**This study**

#### Pseudometarhizium cangyuanense

Taxon classificationFungiSordariomycetesClavicipitaceae

Hong Yu bis, Z.H Liu, H Wang & X.Y Liu sp. nov.

F23F7BB3-CF15-57C5-AB88-12D6BE9B5247

862006

Fig. 3

##### Etymology.

Named after Cangyuan County, where the species were first collected.

##### Type.

Specimens were collected from the *Ophiocordyceps* sp. on 13 August 2025 at an altitude of 1483.9 m, with geographic coordinates 23°13'48"N, 99°18'00"E; by Hong Yu and Pengbin Han; designated as holotype specimen YHH-MCA-20508133 and holotype living culture YFCC08069631.

***Sexual morph***. Sexual morphs were not found.

***Asexual morph***. The stroma of *Ophiocordyceps* sp. with heavy parasitism is embedded beneath the withered leaves, occurring singly or in groups, villous, pale yellowish-green in color, and produces abundant greenish-powdery conidia at the apices (Fig. 3). Colonies on PDA incubated at 25 °C reach 28–31 mm in diameter after 14 days; they are granular, initially villose, later developing white granules, becoming white-green upon sporulation, with white mycelium at the margin and a pinkish-brown reverse. Hyphae are septate and smooth-walled. Phialides arise from the hyphae, solitary, opposite, or verticillate, measuring 7.1–18.4 × 0.8–1.4 μm (x̄ = 8.2 × 1.1 μm, n = 30) at the base and tapering gradually to an apex 0.3–0.9 μm wide, lanceolate in shape. Conidia are rarely solitary, predominantly arranged in chains, hyaline, smooth-walled, and dimorphic, comprising ovoid to subglobose conidia 2.4–3.6 × 1.8–2.6 μm (x̄ = 2.6 × 1.9 μm, n = 30) and fusiform conidia 4.1–7.8 × 1.3–2.2 μm (x̄ = 5.2 × 1.6 μm, n = 30).

##### Host.

*Ophiocordyceps* sp.

##### Known distribution.

Yunnan Province, China.

##### Commentary.

Phylogenetic analyses indicate that *Pseudometarhizium cangyuanense*, *Ps. araneogenum*, and *Ps. lepidopterorum* form a well-supported, monophyletic clade within the genus *Pseudometarhizium* (BS/BPP = 98/1.00). All three species share the characteristic arrangement of fusiform conidia in unbranched chains. However, *Ps. cangyuanense* is distinguished from *Ps. araneogenum* and *Ps. lepidopterorum* by the consistent production of dimorphic conidia – comprising both ovoid and fusiform morphotypes. Morphological comparisons among these taxa are summarized in Table 4. Based on concordant phylogenetic and morphological evidence, *Ps. cangyuanense* is formally described as a novel species of the genus.

**Table 4.** Morphological comparison of asexual morph species of *Pseudometarhizium*.

Species	Host	Synnemata	Phialides	Conidia	References
*Pseudometarhizium araneogenum*	Spider (Araneidae)	Colonies white, consisting of a basal felt, floccose hyphal overgrowth, reverse yellowish to pale brown or green. Prostrate hyphae smooth, septate, hyaline. Erect conidiophores usually arising from aerial hyphae	Phialides solitary or in groups of two, with a cylindrical basal portion, tapering into a short distinct neck	Conidia in chains, hyaline, fusiform, one-celled	Chen et al. (2022)
*Pseudometarhizium lepidopterorum*	Pupa (Lepidoptera)	Colonies, white, consisting of a basal felt and cottony, floccose hyphal overgrowth, reverse yellowish to pale green. Prostrate hyphae smooth, septate, hyaline. Erect conidiophores usually arising from aerial hyphae.	Phialides solitary or in groups of two to three, with a cylindrical basal portion, tapering into a short distinct neck.	Conidia in chains, hyaline, fusiform, one-celled.	Chen et al. (2022)
*Pseudometarhizium cangyuanense*	*Ophiocordyceps* sp. (Ophiocordycipitaceae)	Colonies granular, initially villose, later developing white granules, becoming white-green upon sporulation, with white mycelium at the margin and a pinkish-brown reverse. Hyphae are septate and smooth-walled.	Phialides arise from the hyphae, solitary, opposite, or verticillate at the base, lanceolate in shape.	Conidia are rarely solitary, predominantly arranged in chains, comprising ovoid to subglobose conidia and fusiform conidia.	**This study**

## Discussion

Multilocus phylogenetic frameworks have fundamentally reshaped fungal systematics, particularly within Hypocreales, where reliance on morphology alone has repeatedly proven insufficient for resolving species boundaries and generic placements (Sung et al. 2007; Wang et al. 2021; Xiao et al. 2023). In this study, a survey was conducted to search for and retrieve nuclear gene sequences of the genera *Pseudometarhizium* and *Pleurocordyceps* from the NCBI database. Subsequently, the sequences were compared with the obtained data. In addition, a phylogenetic tree was constructed based on a multi-locus dataset analysis (nr*SSU*, ITS, nr*LSU*, *tef*1-α, *rpb*1, and *rpb*2) to clarify the phylogenetic position of *Pleurocordyceps luopingensis* and *Pseudometarhizium cangyuanense*. Phylogenetically, *Pl. luopingensis* formed an independent clade within the genus *Pleurocordyceps*. Similarly, *Ps. cangyuanense* was closely related to *Ps. araneogenum* and *Ps. lepidopterorum*. However, in terms of morphological characteristics, *Ps. cangyuanense* parasitizes *Ophiocordyceps* sp.; colonies are granular; and both ovoid conidia and fusiform conidia are present. There were also differences in the sizes of phialides and conidia.

The phylogenetic placements observed here contribute to a broader understanding of evolutionary patterns within these genera. The clear separation of the new taxa suggests that species diversity within *Pleurocordyceps* and *Pseudometarhizium* is likely underestimated, particularly in biodiversity-rich regions such as Yunnan Province. This inference aligns with previous studies emphasizing that tropical and subtropical ecosystems harbor cryptic fungal diversity that becomes evident only through multilocus analyses. The present findings therefore reinforce the necessity of molecular phylogenetics for accurately reconstructing lineage diversification within entomopathogenic fungi.

The host association observed in *Ps. cangyuanense* is also biologically noteworthy. Parasitism on *Ophiocordyceps* species suggests complex ecological interactions, potentially involving hyperparasitism or niche specialization. Such relationships are increasingly recognized as drivers of diversification in entomopathogenic fungi and may reflect adaptive strategies linked to resource exploitation or competitive dynamics. Comparable ecological strategies have been documented in other hypocrealean fungi, indicating that host shifts and inter-fungal associations may play a greater role in species evolution than previously assumed. Further investigation into host specificity and life history strategies may therefore yield valuable evolutionary insights.

From an applied perspective, the identification of novel species within these genera has practical implications. Members of *Pseudometarhizium* are closely related to well-known biocontrol fungi and may represent promising candidates for fungal insecticide development. Meanwhile, *Pleurocordyceps* species have attracted attention as sources of bioactive secondary metabolites. Earlier studies demonstrated that *Pleurocordyceps nipponica* produces compounds with antibacterial, antifungal, and antitumor properties (Sangdee et al. 2015, 2016; Thammawat et al. 2017; Surapong et al. 2019). The phylogenetic distinctiveness of *Pl. luopingensis* raises the possibility of lineage-specific metabolite profiles, an aspect warranting future biochemical and genomic exploration. Accurate taxonomic identification thus directly underpins downstream ecological and biotechnological research.

Collectively, this study underscores the taxonomic and phylogenetic significance of integrating multilocus datasets with detailed morphological examination. The recognition of *Pl. luopingensis* and *Ps. cangyuanense* expands current species concepts within these genera and highlights Yunnan Province as an important reservoir of entomopathogenic fungal diversity. Continued sampling and phylogenetic assessment will be essential for clarifying evolutionary relationships, refining species boundaries, and exploring the ecological and applied potential of these fungi.

## Supplementary Material

XML Treatment for Pleurocordyceps luopingensis

XML Treatment for Pseudometarhizium cangyuanense

## Additional information

### Artificial Intelligence (AI) use

The authors accept full responsibility for the content of the manuscript, including the disclosure of any use of AI.

This study used ChatGPT-4 (OpenAI, 2025) to polish the initial draft. All AI-generated content was manually reviewed and modified by the authors.

### Author ORCIDs

Z. Liu https://orcid.org/0000-0003-4118-3694

H. Wang https://orcid.org/0009-0003-1684-5868

X. Liu https://orcid.org/0009-0008-2649-8712

P. Han https://orcid.org/0000-0001-8675-5268

W. Pan https://orcid.org/0009-0006-4752-0849

H. Yu https://orcid.org/0000-0002-2149-5714

### Data availability

All of the data that support the findings of this study are available in the main text.
